# *Openreuma* Consensus on the role of nursing in the care of patients with rheumatoid arthritis and diffuse interstitial lung disease*

**DOI:** 10.17533/udea.iee.v40n3e17

**Published:** 2023-02-14

**Authors:** Laura Cano García, María Jesús García de Yébenes, Natalia Mena Vázquez, José Mª Martín Martín, Carmen Domínguez Quesada, Silvia García-Díaz, Ana Isabel Rodríguez Vargas, Jenny de la Torre-Aboki, Francisco Jiménez Núñez, Francisco Espíldora Hernández, Leticia León Mateos, Ana Vázquez Lojo, Elena Marcos Pérez, Laura Castiblanco, Loreto Carmona

**Affiliations:** 1 Regional University Hospital of Malaga; Malaga, Spain. Regional University Hospital of Malaga Malaga Spain; 2 Instituto de Salud Musculoesquelética (Inmusc); Madrid, Spain. Instituto de Salud Musculoesquelética Madrid Spain; 3 University Hospital Nuestra Señora de la Candelaria; Santa Cruz de Tenerife, Spain. University Hospital Nuestra Señora de la Candelaria Santa Cruz de Tenerife Spain; 4 Virgen Macarena University Hospital; Seville, Spain. Virgen Macarena University Hospital Seville Spain; 5 Consorci Sanitari Integral, L'Hospitalet de Llobregat; Barcelona, Spain. Consorci Sanitari Integral, L'Hospitalet de Llobregat Barcelona Spain; 6 University Hospital of the Canary Islands; Santa Cruz de Tenerife, Spain. University Hospital of the Canary Islands Santa Cruz de Tenerife Spain; 7 Alicante General University Hospital; Alicante, Spain. Alicante General University Hospital Alicante Spain; 8 Hospital Clínico San Carlos; Madrid, Spain. Hospital Clínico San Carlos Madrid Spain; 9 Spanish League Against Rheumatism (LIRE); Spain. Spanish League Against Rheumatism Spain; 10 Moisès Broggi Hospital. San Juan Despí; Barcelona, Spain. Moisès Broggi Hospital Barcelona Spain; 11 OpenReuma; Spain. OpenReuma Spain

**Keywords:** lung diseases, interstitial, arthritis, rheumatoid, consensus, nursing, safety, efficacy., enfermedades pulmonares intersticiales, artritis reumatoide, consenso, enfermería, seguridad, eficacia., doenças pulmonares intersticiais, artrite reumatoid, consenso, enfermagem, segurança, eficacia.

## Abstract

**Objective.:**

To develop practical recommendations, based on the best available evidence and experience, on the nursing management of patients with rheumatoid arthritis (RA) and interstitial lung disease (ILD).

**Methods.:**

The usual consensus methodology was used, with a nominal group, systematic reviews (SRs), and Delphi survey. The expert panel, consisting of rheumatology nurses, rheumatologists, a psychologist, a physiotherapist, and a patient, defined the scope, the users, the topics on which to explore the evidence and on which to issue recommendations.

**Results.:**

Three PICO questions evaluated the efficacy and safety of pulmonary rehabilitation and non-pharmacological measures for the treatment of chronic cough and gastroesophageal reflux by means of SR of the literature. With the results of the reviews, 15 recommendations were established for which the degree of agreement was obtained with a Delphi survey. Three recommendations were rejected in the second round. The 12 recommendations were in patient assessment (*n*=4); patient education (*n*=4); and risk management *(n*=4). Only one recommendation was based on available evidence, while the remaining were based on expert opinion. The degree of agreement ranged from 77% to 100%.

**Conclusion.:**

This document presents a series of recommendations with the aim of improving the prognosis and quality of life of patients with RA-ILD. Nursing knowledge and implementation of these recommendations can improve the follow-up and prognosis of patients with RA who present with ILD.

## Resumen

**Objetivo.:**

Desarrollar recomendaciones prácticas, basadas en la mejor evidencia y experiencia disponible, sobre el manejo de enfermería de los pacientes con artritis reumatoide (AR) y enfermedad pulmonar intersticial (EPI).

**Métodos.:**

Se utilizó la metodología de consenso en la que un panel de expertos (formado por enfermeras de reumatología, reumatólogos, una psicóloga, una fisioterapeuta y una paciente) definió el ámbito, los usuarios, los temas sobre los que explorar la evidencia y sobre los que emitir recomendaciones. Tres preguntas PICO evaluaron la eficacia y seguridad de la rehabilitación pulmonar y las medidas no farmacológicas para el tratamiento de la tos crónica y el reflujo gastroesofágico mediante la búsqueda de revisiones sistemáticas, excluyendo aquellas cuya calidad era baja, muy baja o críticamente baja, según la herramienta AMSTAR-2. Posteriormente, se hizo una reunión para la formulación de recomendaciones que se presentaron con un resumen de la evidencia a la encuesta Delphi.

**Resultados.:**

Con los resultados de las revisiones se establecieron 15 recomendaciones cuyo grado de acuerdo osciló entre el 77% y el 100% en la una encuesta Delphi. Tres recomendaciones fueron rechazadas en la segunda ronda: una por la evidencia disponible y los dos restantes se basaron en la opinión de expertos. Las 12 recomendaciones restantes aprobadas se referían a la evaluación del paciente (*n*=4), a la educación del paciente (*n*=4) y a la gestión del riesgo (*n*=4).

**Conclusión.:**

El conocimiento del consenso *Openreuma* por parte de enfermería y la aplicación sus 12 recomendaciones basadas en la mejor evidencia y experiencia puede mejorar el seguimiento y el pronóstico de los pacientes con AR que presentan EPI.

## Resumo

**Objetivo.:**

Desenvolver recomendações práticas, baseadas na melhor evidência e experiência disponíveis, sobre o manejo de enfermagem de pacientes com artrite reumatoide (AR) e doença pulmonar intersticial (DPI).

**Métodos.:**

Foi utilizada a metodologia de consenso, com grupo nominal, revisões sistemáticas e levantamento Delphi. O painel de especialistas, formado por enfermeiros reumatologistas, reumatologistas, psicólogo, fisioterapeuta e paciente, definiu o escopo, os usuários, os tópicos sobre os quais explorar as evidências e sobre os quais emitir recomendações. Três questões do PICO avaliaram a eficácia e segurança da reabilitação pulmonar e medidas não farmacológicas para o tratamento da tosse crônica e refluxo gastroesofágico por meio de RS. Aqueles cuja qualidade era baixa, muito baixa ou criticamente baixa, de acordo com a ferramenta AMSTAR-2, foram excluídos. Posteriormente, realizou-se uma reunião para formular recomendações que foram apresentadas com um resumo das evidências ao inquérito Delphi.

**Resultados.:**

Com os resultados das revisões, foram estabelecidas 15 recomendações cujo grau de concordância entre 77% e 100% foi obtido com uma pesquisa Delphi. Três recomendações foram rejeitadas na segunda rodada. As 12 recomendações referiam-se à avaliação do paciente (*n*=4); à educação do paciente (*n*=4); e ao gerenciamento de risco (*n*=4). Apenas uma recomendação foi baseada nas evidências disponíveis, enquanto as demais foram baseadas na opinião de especialistas.

**Conclusão.:**

Este documento apresenta uma série de recomendações com o objetivo de melhorar o prognóstico e a qualidade de vida dos pacientes com AR-ILD. O conhecimento da enfermagem e a aplicação dessas recomendações podem melhorar o acompanhamento e o prognóstico de pacientes com AR com DPI.

## Introduction

The term "diffuse interstitial lung disease" (ILD) encompasses a heterogeneous group of diseases with common clinical, radiological, and histological features that may occur in association with autoimmune processes, such as rheumatoid arthritis (RA), or without known cause. Although a recently published prevalence of ILD in RA of around 5% has been reported, the prevalence, as well as the clinical and histological characteristics of these diseases show large variability.(1) Differences are due to, among other reasons, its often-subclinical nature, the different populations studied, and the diagnostic methods used. Risk factors for the development of ILD in RA include older age, male sex, smoking, and seropositivity for rheumatoid factor and anti-cyclic citrullinated peptide antibodies.(2) The predominant symptoms of ILD are exertional dyspnoea and chronic dry cough. In addition, up to 50% of patients also have gastro-oesophageal reflux disease (GORD).(3) ILD is one of the main causes of morbidity and mortality in RA, with an estimated median survival of 3-7 years, comparable to some neoplastic diseases.(1,3) Dyspnoea and cough cause significant functional impairment which, together with poor prognosis, favours the onset of depressive symptoms and loss of quality of life.(4,5)

The recommendations of the Spanish Society of Rheumatology (SER) and the Spanish Society of Pneumology and Thoracic Surgery (SEPAR) establish the need for multidisciplinary therapeutic management in patients with RA-associated ILD,(6) an aspect that has been repeatedly pointed out by several authors.(3,7) In fact, the *National Institute for Health and Care Excellence* (NICE) has underlined the importance of specialist nursing in the management of patients with idiopathic pulmonary fibrosis, a type of ILD, as have the European Alliance of Associations for Rheumatology (EULAR) recommendations for the management of patients with RA-associated ILD and also the 2018 EULAR recommendations on the role of nursing in the management of chronic inflammatory arthritis.(8,9) Responsibilities may include: patient and family education, assessment of symptoms (dyspnoea, fatigue, cough, and psychological distress) and comorbidities, coordination with other healthcare professionals, participation in research projects, addressing questions about severity, progression, diagnostic tests and pharmacological and non-pharmacological treatment options, and advice on support groups and other resources.(4,5,7) 

Despite the importance of multidisciplinary treatment, the reality is that there are no practical guidelines on the specific aspects that nursing professionals face on a daily basis in the management and follow-up of patients with PIDD. Nurses assessing patients with RA-ILD are part of a healthcare team in close collaboration with the patient and family/significant others, the rheumatologist and pulmonologist, both ideally located in multidisciplinary units. Unfortunately, at present there is no speciality in rheumatology or pulmonology nursing. With these considerations in mind, OpenReuma has promoted the development of a document of recommendations, based on evidence or expert opinion, to help nurses improve the management and follow-up of patients with PIDD.

## Methods

The usual methodological approach for evidence-based consensus was used, including nominal group, systematic review (SR), and Delphi survey. The study was conducted in the following successive phases: 1) nominal group with an expert panel and patient interview; 2) SRs; 3) consensus meeting for the development of recommendations; and 4) Delphi voting on the degree of agreement. By its nature, the project was exempt from the need for approval by a research ethics committee. However, it was guided in accordance with the principles set out in the Declaration of Helsinki, applicable Good Clinical Practice regulations and current legislation on confidentiality. 

### Nominal group and panel of experts

To address all aspects of interest, a multidisciplinary panel of 13 people - 7 rheumatology nurses, 2 rheumatologists, 1 pulmonologist, 1 psychologist, 1 physiotherapist and occupational therapist and 1 patient with ILD - was formed. In a first meeting of the panel, the guidelines of the document, the scope, the users, and the structure were defined. In addition, the points on which to explore the evidence were identified. The panel met twice and was kept informed throughout the development of the project via the Miro® platform.

### Literature review

The clinical questions defined by the panel were transformed into the PICOt epidemiological format so that they could be answered by systematic literature review (Table 1). We evaluated the efficacy and safety of pulmonary rehabilitation and non-pharmacological interventions to improve two typical problems of ILD: refractory cough and GORD.

**Table 1:** PICOts used in the systematic reviews

Question	Population	Intervention	Comparator	Outcome	Type of study
Is pulmonary rehabilitation effective in ILD?	ILD	Pulmonary rehabilitation	Pharmacological, non-pharmacological treatments and ineffective or sham interventions	Effectiveness and safety	SRs and RCTs
What is the efficacy of non-pharmacological interventions for refractory cough?	Refractory chronic cough	Non-pharmacological interventions	Pharmacological, non-pharmacological treatments and ineffective or sham interventions	Effectiveness and safety	SRs and RCTs
What is the efficacy of non-pharmacological interventions in gastro-oesophageal reflux?	GORD	Non-pharmacological interventions	Non-pharmacological treatments and ineffective or sham interventions	Effectiveness and safety	SRs and RCTs

Abbreviations: ILD, interstitial lung disease; GORD, gastro-oesophageal reflux diseases; SRs, systematic reviews. RCTs=randomised clinical trials.

The reviews conducted were hierarchical, i.e., for each question, existing SR papers were first identified and assessed for bias. We only proceeded to search for primary studies in cases where the evidence was not sufficiently robust, direct, and consistent to answer the question posed. A search strategy was established for each question including terms related to ILD, pulmonary rehabilitation, refractory cough, or GORD, both MeSH and free text, filtered by study type "systematic review" (Table 2). Articles were peer-selected by title and abstract using Rayyan® software and then read in detail to check for eligibility. 

**Table 2:** Search strategies

PICOt	Terms
ILD	Lung Diseases, Interstitial )MeSH Terms). Pulmonary Fibrosis (MeSH Terms). “Diffuse Parenchymal Lung Disease”(Text Word). Interstitial (Text Word) AND lung Text( Word) AND disease* (Text Word). Pneumon*(Text Word) AND Interstitial(Text Word) (pulmonary*(Text Word) OR lung*(Text Word) OR alveoli*(Text Word) AND (fibros*(Text Word) OR fibrot*(Text Word)
Pulmonary rehabilitation	(rehabilitat*(Text Word) OR fitness*(Text Word) OR exercis*(Text Word) OR physical*(Text Word) OR train*(Text Word) OR activ*(Text Word) OR physiotherap*(Text Word) OR kinesiotherap*(Text Word) OR exert*(Text Word) OR "Physical Therapy Modalities" (MeSH) OR "Exercise" (MeSH) OR "Physical Fitness" (MeSH) OR "Physical Exertion" (MeSH) OR "Rehabilitation" (MeSH)
GORD	"Gastroesophageal Reflux"(Mesh). (gastric AND acid AND reflux) (gastro-esophageal OR gastro-oesophageal OR gastro-oesophageal OR gastroesophageal) AND (reflux disease) GERD
Cough	"Cough(Mesh)"(Mesh (chronic OR subacute OR SUB-ACUTE) AND cough refractory AND cough cough.
SR	(systematic review(ti) OR systematic literature review(ti) OR systematic scoping review(ti) OR systematic narrative review(ti) OR systematic qualitative review(ti) OR systematic evidence review(ti) OR systematic quantitative review(ti) OR systematic meta-review(ti) OR systematic critical review(ti) OR systematic mixed studies review(ti)OR systematic mapping review(ti) OR systematic mapping review(ti) OR systematic cochrane review(ti) OR systematic search and review(ti) OR systematic integrative review(ti)) NOT comment (pt) NOT (protocol (ti) OR protocols (ti))) NOT MEDLINE (subset) Cochrane Database Syst Rev (ta) AND review (pt)) OR systematic review (pt)

Abbreviations: ILD, interstitial lung disease; GORD, gastro-oesophageal reflux diseases; SR, systematic review.

The methodological quality of the reviews selected was assessed using the AMSTAR-2 tool, excluding those whose quality was low, very low, or critically low.(10) If the evidence was not sufficiently solid, direct, and consistent to answer a specific question, a search for primary studies was carried out. The methodological assessment was performed using the Cochrane Rob 2 risk of bias tool and Jadad's scale for the risk of bias.(11,12) In order to facilitate informed decisions by the panel, tables including information from the selected studies were prepared using the GRADE methodology.(13) For this purpose, the most relevant results were selected and the level of evidence for each specific question was assigned. The GRADE system classifies the quality of evidence into four levels: high, moderate, low, and very low.

### Meeting for the formulation of recommendations

Once the literature review was completed and based on the issues raised at the first meeting, the steering group produced a draft of the recommendations to work on. These recommendations, together with their evidence, were presented at a second meeting of the panel for discussion and consensus editing. For each of the proposed recommendations a first vote was taken during the meeting. In this first vote, only those recommendations were voted for or against. Consensus was only reached for those recommendations that achieved 65% in favour. Adjustments were then made to the wording to reflect all the panel's perspectives. In addition, recommendations not proposed by the steering group were added during the meeting, following the same methodology. All the panel's discussions were recorded in minutes that served as the basis for the final document.

### Assessment of the degree of agreement: Delphi survey

The recommendations obtained at the consensus meeting were transformed into the items of a Delphi survey. This Delphi was answered by the panel members and sent to the OpenReuma members (rheumatologists and nurses), as potential users of the recommendations. In the Delphi (conducted with Welphi®), each item was presented with a summary of the evidence. The degree of agreement was scored on a scale from 0 (strongly disagree) to 10 (strongly agree). In a first round, corrections to the text were allowed. If a recommendation did not require corrections and reached more than 75% agreement, it was not passed to the second round.

## Results

**Figure 1:**
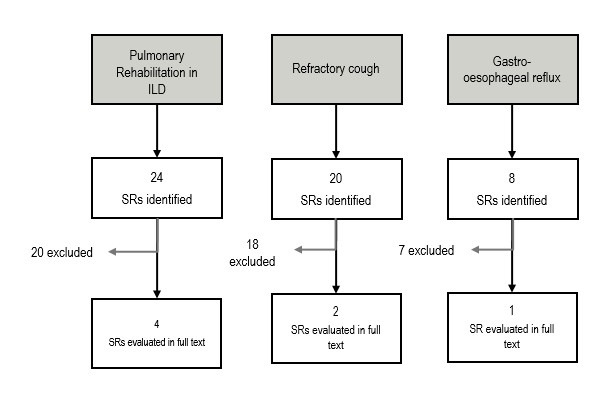
Flow chart of systematic reviews *Abbreviations: ILD, interstitial lung disease; SR, systematic review.*

The results of the SRs are presented for each research question: 1) Efficacy of pulmonary rehabilitation in ILD. Twenty-four SRs were identified of which 4 were finally included for detailed reading; 2) Efficacy of non-pharmacological interventions for the treatment of refractory cough. This search was not narrowed down by ILD, and therefore included refractory cough of any cause. Twenty SRs were identified, of which two were finally included for detailed reading; and 3) Efficacy of non-pharmacological interventions for the treatment of GORD. This search was also not narrowed by ILD and therefore included reflux of any cause. Eight SR were identified, of which only one was selected for detailed reading, although it did not include patients with GORD. The flow chart of the three research questions is presented in Figure 1. 

A total of 15 recommendations were formulated for which a summary of the evidence obtained is presented. Table 3 shows the complete list of recommendations with their level of evidence according to GRADE and the degree of agreement.

Only one of the 12 recommendations was based on the SRs, while the rest are based on expert opinion, although they are easy to justify.

**Table 3:** Recommendations for the management of patients with RA-ILD by the rheumatology nurse

Number	Recommendation	Level of evidence^*^	Agreement^†^
1	Screening for comorbidities should be performed.	NA	81%
2	The nurse should assess and screen for signs and symptoms of ILD in patients with RA.	NA	86%
3	Adherence to treatment should be assessed on a regular basis.	NA	100%
4	The nurse should assist in regular monitoring of treatment safety.	NA	100%
5	The patient should be educated in the early detection of treatment-related adverse effects.	NA	100%
6	ILD-specific aspects should be included in the health education programme for patients with RA.	NA	95%
7	The patient should be counselled and supported in smoking cessation.	NA	100%
8	The rheumatology nurse should educate the patient on infection prevention and identification.	NA	90%
9	In case of reflux or orthopnoea, it may be recommended that the head of the bed be raised.	Very low	95%
10	It is recommended to complement the assessment of patients with ILD with specific PROMs.	NA	95%
11	If frailty is suspected, it should be confirmed by a validated instrument.	NA	86%
12	The nurse should identify available resources for referral of complicated psychosocial cases.	NA	77%

Abbreviations: ILD, interstitial lung disease; RA, rheumatoid arthritis; NA, not applicable; PROMs, patient-reported outcomes. * GRADE (Very High, High, Moderate, Low, Very Low). Not applicable (NA) when there is no systematic review, but it is based on expert consensus; ^†^ After the second Delphi round.

R1. Screening for comorbidities should be performed. Patients with RA should have access to a nurse with knowledge in rheumatic diseases and related diseases, among which ILD is a very relevant one. RA is a chronic inflammatory disease with significant associated comorbidity that has a major impact on patients' functional status, outcome, and quality of life.(14,15) Therefore, comorbidity management in these diseases is of particular importance. Consensus documents have been developed with specific recommendations for the assessment and management of comorbidity in RA,(16) although the pressure of care may be a limiting factor in following these recommendations. To facilitate the management of comorbidity in these patients, specific checklists can be used, both for healthcare professionals and for the patients themselves.(17) The role of nursing in comorbidity management has been emphasised by several authors,(16,18,19) as well as by the EULAR recommendations on the role of nursing in chronic inflammatory arthritis published in 2018.(9)

R2. The nurse should have the training to assess and screen for signs and symptoms of ILD in patients with RA. The SER-SEPAR recommendations on ILD state that patients with RA and respiratory symptoms or auscultation of velcro-like crackles should be systematically screened for ILD.(6) In addition, the EULAR recommendations for nurses stress that "some tasks, traditionally performed by rheumatologists and physiotherapists, such as joint examination, and assessment of signs and symptoms can be learned and performed by nurses with minimal training".(9) It would therefore be advisable to implement nursing training for the correct identification of signs and symptoms associated with ILD, e.g., perform pulmonary auscultation, and interpret the meaning of respiratory function tests. Assessment of dyspnoea by nurses is a simple task that is performed quickly and increases the confidence of the nurse by improving the efficiency of the patient-centred model. Nurses are aware of the importance of routinely measuring breathlessness and standardising this process.(20) It is recommended to use the modified Medical Research Council (mMRC) scale, a very simple scale consisting of 5 levels ranging from 0 (no dyspnoea) to 5 (disabling dyspnoea).(21) 

R3. Adherence to treatment should be assessed on a regular basis. Patient education improves adherence to treatment.(9) Although there are no specific recommendations on the type of intervention to be applied to improve adherence, the EULAR recommendations on adherence to treatment state that all professionals involved in the management of patients with rheumatic and musculoskeletal diseases should promote adherence to treatment and use tailored strategies.(22) Similarly, they insist on the need to assess adherence on a regular basis, based on open-ended questions, especially when the disease is not well controlled. In these recommendations there are useful examples and checklists to adapt to the rheumatology nursing practice.

R4. The nurse must collaborate in the periodic monitoring of treatment safety. Nursing plays a key role in pharmacovigilance activities, improving patient safety and reducing treatment costs. The nurse is the healthcare professional who administers the medicines and usually follows the patient's progress first-hand and is therefore able to identify possible adverse reactions. However, the reporting of adverse reactions is exceptionally low.(23) The results of a SR have shown that, despite the positive attitude of nursing professionals towards pharmacovigilance activities, their level of knowledge and practice lacks adequate competence, with lack of training being the most important obstacle;(24) therefore, continued training of professionals to become competent in pharmacovigilance activities should be ensured, and qualitative studies aimed at discovering new ways to improve involvement in these processes should be promoted.

R5. The patient should be educated in the early detection of adverse effects resulting from treatment. The first EULAR recommendation on the role of nursing in chronic inflammatory arthritis states that patients should have access to a nurse for needs-based education to improve knowledge and management of their disease.(9) 

R6. ILD-specific aspects should be included in the health education programme for patients with RA. Patient education should fulfil different activities aimed at aspects related to health promotion, abandonment of harmful habits, treatment, warning signs of complications, exercise, to foster patient independence and self-management of their disease. Educational activities are applicable at the individual level, e.g., through the use of PROMs (Patient-reported outcome measures), or at the group level and should also include family members. Educational activities should be coordinated between the pulmonology and rheumatology units.

R7. The patient should be counselled and supported in smoking cessation. Several studies have identified smoking as a risk factor for the development of ILD, although no direct relationship with mortality has been observed.(2,25-28) Smoking cessation counselling is of paramount importance in these patients.(3) In this regard, it is important to highlight the usefulness of the smoking cessation units that exist in the various Spanish autonomous communities. These units are linked to hospitals and primary care teams and their coverage varies from one region of Spain to another. On the other hand, the guidance and monitoring of patients by nurses with specific training can be useful, although the evidence on the effectiveness of behavioural support, provided by nurses, to motivate and maintain abstinence from smoking is moderate.(29) 

R8. The rheumatology nurse must educate the patient on infection prevention and identification. Patients with ILD are at increased risk of infection due to a combination of factors, such as the lung disease itself, immunosuppressive treatment, and immune system alterations,(2,4) and is a major cause of mortality.(1) It is therefore essential to maintain vigilance for warning signs or suspected infection and to encourage routine vaccination against influenza and pneumococcus, as established in the SER-SEPAR recommendations.(6) Preventive measures against infection are essential in patients’ protection. The nurse can reuse information on the internet, such as that available on MedlinePlus (https://medlineplus.gov/spanish/infectiousdiseases.html) or the American CDC (https://www.cdc.gov/ncbddd/spanish/sicklecell/healthyliving-prevent-infection.html), or request brochures from the hospital's Preventive Medicine Service to have available for consultation. Vaccination is usually performed at primary care following recommendations made by pulmonologists or rheumatologists.

R9. In case of reflux or orthopnoea, it may be recommended that the head of the bed be elevated. The existence of a link between GORD and various lung diseases underlines the importance of using measures that can reduce reflux with its possible effect on dyspnoea and the occurrence of exacerbations.(30,31) A SR was found on the effect of head-of-bed elevation in patients with GORD, although it did not specifically include patients with ILD or RA. The results of this article showed that elevating the head of the bed, or sleeping with a wedge under a pillow, improves GORD symptoms, with a very limited impact on the reduction of reflux episodes. However, this review has an elevated risk of bias and high heterogeneity, thus a very low level of evidence.(32)

R10. It is recommended to complement the assessment of patients with ILD by specific PROMs. PROMs are objective measures of the patient's perception of different aspects of the disease. Since the impact of ILD on the patient's life is so important, the use of PROMs is essential to obtain a true approximation of the impact of the disease and its treatments from the patient's perspective. In addition, they are especially useful to improve physician and patient satisfaction, efficiency, communication, as well as decision making. In ILD, PROMs can be used primarily to assess symptoms (dyspnoea, cough, fatigue), quality of life, and impact of treatment. Symptom assessment, such as dyspnoea, can be done with the modified Medical Research Council (mMRC) scale, as previously discussed.(21) With regard to quality of life, validated questionnaires for patients with ILD, such as the Saint George Respiratory Questionnaire (SGRQ-I),(33) or the King's Brief Interstitial Lung Disease Questionnaire (K-BILD) can be used.(34) Finally, the psychological impact can be measured using the Hospital Anxiety and Depression Scale (HADS), or the Beck Depression Inventory (BDI).(35) A review on the use of PROMs in ILD, published in 2021, affirms the need to use instruments specifically designed for ILD and properly validated, and also sets out a series of recommendations for their use.(36) 

R11. If frailty is suspected, it should be confirmed by a validated instrument. Frailty is defined as a physiological state of increased vulnerability to stressors due to decreased physiological reserves. Cohort studies have shown that about half of all patients with ILD have frailty and its importance lies in its predictive role in disability, hospitalisation, and mortality.(37,38) Two models of frailty have been described; Fried's model based on biomedical factors,(39) and the Rockwood's model based with a more holistic definition including psychosocial and environmental factors.(40) Assessment of frailty is important for the possibility of intervention. However, although multiple instruments exist, not all of them are validated. Fried's model is the most widely accepted and defines frailty by the presence of at least 3 of the following criteria: unintentional weight loss of 4.5 kg in the last year, subjective feeling of exhaustion (feeling unusually tired in the last month), weakness with objective lack of strength, decreased walking speed and low physical activity. The consensus document on frailty and falls prevention in the elderly, of the Ministry of Health and Social Affairs of 2014 proposes the SHARE-FI scale,(41) validated in the Spanish population, based on the Fried criteria and applicable to non-institutionalised patients.(42) There are free access calculators for this scale, differentiated by sex, at https://sites.google.com/a/tcd.ie/share-frailty-instrument-calculators/. On the other hand, a scale has been developed in Spain to measure the biological characteristics of frailty, The Frailty Trait Scale (FTS), based on Fried's model, although it also incorporates the framework proposed by Rockwood. It consists of 12 items grouped into 7 dimensions: energy balance-nutrition, physical activity, nervous system, vascular system, strength, endurance, and walking speed. This scale has predictive value for mortality in people over 80 years of age, and for hospitalisations in people under 80.(43) 

R12. The nurse must identify available resources for referral of complicated psychosocial cases. Appropriate management of psychosocial problems improves health outcomes and quality of life for patients. The nurse is one of the closest professionals to the patient, and therefore plays a fundamental role in the detection and referral of those with psychosocial problems. 

## Discussion

This paper presents a series of recommendations, based on the best available evidence and the opinion of a multidisciplinary group of experts, to assist nursing professionals in the management and follow-up of patients with RA-ILD. In general, existing guidelines or recommendations, such as those of SER-SEPAR, are purely clinical and do not include specific information for nurses. On the other hand, the high complexity of patients with RA-ILD requires adapting the available evidence to the context of the rheumatology nurse by developing specific recommendations to improve patient management in complex situations in which the nurse can play a role. With these considerations, twelve recommendations for the management of patients with RA-ILD by the rheumatology nurse have been established. Four recommendations are about assessment (identification), four about education (prevention), and four about of risk management (detection). Only one of the recommendations was based on available evidence, with a very low level of evidence; the rest were based on expert opinion, so the level of evidence does not apply. The degree of agreement of the recommendations ranged from 77% to 100%. 

The assessment of possible lung involvement in RA patients is very important, as the occurrence of ILD carries a worse prognosis and should be done in a multidisciplinary way (integrated clinical diagnostic model).(6) This is the setting for recommendations R1 (screening for comorbidities), R2 (assess and screen for signs and symptoms of ILD in patients with RA), R10 (use of specific PROMS), and R11 (assessment of frailty). Patient education is a key tool to promote self-management, self-efficacy, and appropriate coping with the disease in order to facilitate independence,(44) and a major role of nursing. This aspect is reflected in recommendations R5 (education to detect adverse events), R6 (education on specific aspects of ILD), R7 (importance of smoking cessation), and R8 (need to identify and prevent infections). 

Risk management represents the set of pharmacovigilance activities and interventions designed to identify, characterise, and prevent or minimise the risks of medicines and to evaluate the effectiveness of these interventions. Risk management can occur at different points during treatment: before treatment initiation, during follow-up, and in the assessment of potential undesirable effects that may occur.(45) Two recommendations address this aspect, R3 (referring to the need to regularly assess adherence to treatment), and R4 (on the collaboration of the nurse in the periodic monitoring of treatment safety). Recommendation R9, referring to the efficacy of the bedside in case of reflux, refers to the effectiveness of an intervention to reduce risk of complications and was the only evidence-based recommendation, although with a very low level according to GRADE. Recommendation R12, referring to the identification and referral of patients with psychosocial problems, belongs to the context of risk management in special situations. 

The following three recommendations were rejected in the second round of the Delphi:

- “The nurse must have a basic knowledge of oxygen therapy and CPAP in order to be able to answer the patient's questions”.
- “Patient access to respiratory rehabilitation units should be facilitated”; this recommendation had 71% agreement and was evidence-based, although with a very low level of evidence.
- “In case of refractory cough, in addition to pharmacological measures, speech rehabilitation may be considered”; the level of agreement for this recommendation was very low, 59%, although it was evidence-based, with a very low level of evidence.

Unfortunately, the Delphi panel was very clear in excluding these recommendations. We cannot but reflect what happened, given that the methodology was established a priori and approved by all. Among the limitations of this work, it should be noted that all but one of the recommendations were based on expert opinion due to the lack of specific studies. In addition, it is important to note that the recommendations rejected in the second round were evidence-based, although their level of evidence was very low. One explanation for the lack of studies on the topics of the recommendations may have to do with the majority of resources being allocated to projects related to the therapeutic efficacy of specific drugs. On the other hand, the rejection of the three aforementioned recommendations could be related to the lack of specific training in ILD by nurses and the belief of overlapping roles with other health professionals. The main strength of this study is the participation of a multidisciplinary team of professionals with extensive experience in the management of patients with RA-ILD and a great interest in this topic. 

In conclusion and considering the fundamental role of nursing in the management and follow-up of patients, this document presents a series of recommendations to improve the health outcomes of patients with RA-ILD. Nursing knowledge and implementation of these recommendations can facilitate the follow-up and improve the prognosis of patients with rheumatoid arthritis who present with diffuse interstitial lung disease.
